# Individual Characteristics Influencing Physicians’ Perceptions of Job Demands and Control: The Role of Affectivity, Work Engagement and Workaholism

**DOI:** 10.3390/ijerph13060567

**Published:** 2016-06-06

**Authors:** Greta Mazzetti, Roberta Biolcati, Dina Guglielmi, Caryn Vallesi, Wilmar B. Schaufeli

**Affiliations:** 1 Department of Educational Science, University of Bologna, Via Filippo Re, 6-40126 Bologna, Italy; r.biolcati@unibo.it (R.B.); dina.guglielmi@unibo.it (D.G.); caryn.vallesi@studio.unibo.it (C.V.); 2 Department of Psychology, Utrecht University, P.O. Box 80.140, 3508 TC Utrecht, The Netherlands; w.schaufeli@uu.nl; 3 Department of Psychology, University of Leuven, 3000 Leuven, Belgium

**Keywords:** affectivity, work engagement, workaholism, job control, job demands, JD-R Model

## Abstract

The first purpose of the present study was to investigate the role of individual characteristics, *i.e.*, positive and negative affectivity, in explaining the different perception of job control and job demands in a particularly demanding environment such as the healthcare setting. In addition, we aimed to explore the mediational role of work engagement and workaholism using the Job Demands-Resources Model as a theoretical framework. Data were collected using a sample of 269 Italian head physicians working in nine general hospitals. To test our hypotheses, the collected data were analyzed with structural equation modeling. Moreover, Sobel Test and bootstrapping were employed to assess the mediating hypotheses. Our results indicated that positive affectivity is related to work engagement, which, in its turn, showed a positive association with job control. In addition, workaholism mediated the relationship between negative affectivity and job demands. All in all, this study represents a first attempt to explore the role of trait affectivity as a dispositional characteristic able to foster the level of work engagement and workaholism exhibited by employees and, in turn, to increase the perceived levels of job control and job demands.

## 1. Introduction

Past research has focused on job characteristics that impact both positive and negative work-related outcomes. For instance, demanding aspects of one’s job such as work pressure, emotional demands, and role ambiguity were found to relate significantly with job burnout [1] and psychological health symptoms [2]. In contrast, positive job-related aspects, e.g., perceived organizational support and transformational leadership, showed significant associations with positive outcomes, e.g., job performance [3] and job satisfaction [4]. Overall, perceived job characteristics have been conceived as antecedents of employees’ attitudes and behaviors. For instance, the Job Demands-Resources (JD-R) model [5] identified two broad categories of job characteristics as major antecedents of employees’ strain (e.g., job burnout) and motivation (e.g., work engagement), these categories refer to job demands and resources. The JD-R model has been applied extensively to the study of the influence of job demands and resources on a wide range of organizational and individual outcomes. Although the original version of the JD-R model only included job characteristics, it has been subsequently extended by integrating individual characteristics in terms of personal resources [6]. Empirical evidence suggests that individual dispositions may significantly impact on work engagement and workaholism, which represent two opposite kinds of heavy work investment [7,8]. In contrast, research on the role played by personal dispositions in predicting different perceptions of job characteristics is far from being exhausted [9].

Consistent with the bottom-up theory, individual characteristics such as trait affectivity have been shown to predispose individuals to different attitudes towards their job [10,11]. Affectivity is defined as an emotion-based trait dimension that determines a cognitive bias through which individuals address the experiences and may influence how they live and evaluate their jobs [12].

The present research aims at exploring individual characteristics that may explain differences in perceptions of job demands and control among employees working in the same environment—which in the current study is a healthcare setting. This work setting is widely recognized as particularly demanding and physicians have long been considered to be at an occupation at risk for the development of burnout and distress symptoms, such as anxiety and depression [13]. In particular, physicians have to deal with job demands, such as time pressure, role ambiguity, and conflicting relationships with colleagues and patients' families [14]. In addition, and perhaps most importantly, physicians’ perception of their job demands and resources has shown to affect not only their health and well-being, but also the quality of medical care they provide [15].

### 1.1. The Influence of Trait Affectivity on Opposite Kinds of Working Hard

The influence of dispositional traits on individual perceptions and attitudes has been well-established by a large amount of empirical results [16]. Among these traits, affective dispositions have been shown to be stable over time and across situations [17]. For the most part, research on affectivity was driven by the seminal paper of Watson and colleagues [12], that identified positive and negative affectivity as basic dimensions of the affective experience: individuals with high positive affectivity are characterized by energy, enthusiasm and optimism, whereas those with high negative affectivity are characterized by distress, nervousness and pessimism. In line with this description, positive trait affectivity has been explored in empirical investigations assessing the individual antecedents of the positive affective-motivational state identified as work engagement.

To be specific, work engagement is defined as a work-related state of fulfillment that consists of three interrelated dimensions: vigor, dedication, and absorption [18]. According to this definition, vigor entails high levels of energy and mental resilience while working, the willingness to invest effort in one’s work, and perseverance in the face of difficulties; dedication is defined as being involved in one’s work, and experiencing a sense of enthusiasm, inspiration, pride, and challenge; and absorption is described as being happily engrossed in one’s work, so that time passes quickly and one has difficulties detaching from work. The positive nature of work engagement is substantiated by its association with several positive outcomes: for instance, engaged employees show greater organizational commitment and enhanced job performance [2], are more satisfied with their jobs [19], and exhibit higher levels of proactivity [20] and extra-role behavior [21]. A large body of empirical evidence points out that engaged employees are primarily driven by a so-called autonomous motivation [22]. This type of motivation promotes intrinsically motivated behavior, whereby activities are carried out for their own sake [23]. Accordingly, engaged employees invest an extraordinary amount of time in their work because they truly enjoy this activity and are happily engrossed in it.

Work engagement has been described as a psychological state specific of the work domain that is strongly affected by positive affectivity, since this dispositional trait predicts general affective tendencies across different life domains [24]. Indeed, positive affectivity markers such as attentive, alert, enthusiastic, inspired, proud, determined, energized, strong, and active are included in the Positive Affectivity scale of the PANAS [12]. These descriptors implicate affective states that are highly compatible to the aforementioned dimensions of engagement. Thus, employees characterized by positive affectivity are dispositionally more prone to experience a positive form of involvement in their work, so they are more engaged in this activity [25]. Langelaan and colleagues [26] provided empirical support to this assumption by showing a positive association between work engagement and extraversion, an indicator of positive affectivity entailing an individual disposition towards cheerfulness, sociability, and high activity [27]. Work engagement has been shown to relate significantly not only to dispositional positive affectivity, but also to a pattern of affect regulation that allow engaged employees to also shift promptly to a positive mood after having encountered tricky situations at work [28]. In addition, positive affectivity is also strongly related to engagement through the improvement of employees’ capability to translate an intention into action and to identify suitable goals, thus supporting the process of goal-directed action [29]. Overall, these findings reveal that individual disposition toward the occurrence of positive affective states plays a significant role in experiencing a work-related state of fulfillment that underlies work engagement. Accordingly, the following hypothesis was tested:

*Hypothesis 1*. Positive affectivity is associated to higher levels of work engagement.

The tendency to work hard and display a great level of dedication to one’s job represents a significant point of overlap between engagement and a negative type of working hard, *i.e.*, workaholism. Workaholism represents a negative psychological state characterized by working excessively and in a compulsive manner [30]. According to this definition, working excessively represents the behavioral dimension of the construct and implies that the amount of time and energy that workaholic employees devote to their work exceeds any request what would be indispensable to fulfill organizational or economic requirements. Working compulsively, on the other hand, constitutes the cognitive component of workaholism and indicates that these employees are obsessed with their work and persistently think about activity, even when they are not working. Previous studies have consistently suggested that workaholism leads to detrimental effects on different life domains. With reference to one’s work, workaholic employees have been shown to display an impaired work performance [31], and to report conflicting relationships with their colleagues [32]. Given the extraordinary amount of time dedicate to work-related activities, workaholic employees have insufficient time for recovery and impaired social relationship outside work [33], and a higher incidence of marital problems [34]. In addition, workaholism may negatively affect employees’ health and well-being. Indeed, this negative form of working hard has been found to predict mental distress and health complaints [35,36] and it is associated to higher levels of exhaustion [37]. During the last decade, several perspectives on workaholism have been developed that suggest that this addiction to work originates from the joint impact of person characteristics and environmental factors [38]. Accordingly, Mazzetti and colleagues [39] observed high levels of workaholism when employees both possessed person characteristics that predispose them towards this compulsive behavior and perceived an overwork climate in their workplace, thus an organizational environment that require them to devote an extraordinary amount of time and energy to their work.

In contrast to work engagement, workaholic employees are primarily driven by a controlled motivation [40]. Hence, they strive to avoid disapproval by others and, at the same time, to obtain appreciation. The adoption of external standards of self-worth and social approval without a whole identification with them results in incessant attempts to meet these standards and, in turn, experience a sense of self-esteem: the failure in reaching these standards may lead to the experience of negative emotions and self-criticism [41].

Wojdylo and colleagues [42] argued that the main mechanism of work addiction is the compensatory function of emotions, which explains the inner drive of workaholic employees in fulfilling unrealistic standards of perfectionism. Several studies recognized in obsessive perfectionism the need to compensate for low self-worth and to avoid further negative feelings through compulsive working [43]. The behavioural dimension of workaholism, that is working excessively, may be interpreted as an individual strategy employed in order to prevent employees from experiencing negative emotions and painful feelings of inadequacy. Accordingly, it may be argued that workaholic employees use the act of working as a means for regulating their trait negative affectivity [41]. Moreover, negative affectivity has been defined as a significant antecedent of workaholism [38]. Empirical evidence corroborated this assumption by showing a strong association between negative affectivity and this addiction to work [44]. Specifically, it has been suggested that workaholic employees work so hard in order to avoid the experience of negative affective states [45]. Thus, the following study hypothesis was formulated:

*Hypothesis 2*. Negative affectivity is positively related to higher levels of workaholism.

### 1.2. Different Perceptions of Job Demands and Resources: Assuming an Individual Perspective

Taken together, the empirical evidence discussed above suggests that dispositional traits such as positive and negative affectivity may boost opposite forms of heavy work investment, *i.e.*, work engagement and workaholism. This may be conceptually framed into the Job Demands-Resources (JD-R) Model [5]. As previously mentioned, the JD-R model assumes that employees’ well-being stems from a wide range of workplace characteristics that can be conceptualized as either job demands (*i.e.*, characteristics of the job that require effort and are therefore associated with physiological and psychological costs) or job resources (*i.e.*, those job-related aspects that allow employees to cope with the demanding aspects of their job and simultaneously stimulate them to learn from and grow in it) [46]. Excessive job demands and lacking job resources exert an energy-draining effect on employees through a so-called health impairment process, whereas high levels of job resources may lead to positive work-related and individual outcomes through a motivational process. In addition to job demands and resources, there is compelling evidence suggesting that also dispositional characteristics represent personal demands and resources able to play a significant role as initiators of the JD-R model processes. On the one hand, personal resources such as self-efficacy and optimism contribute in explaining variance in employees’ strain and work engagement [8]. On the other hand, there is substantial evidence that personal aspects such as overcommitment and deficit in the cognitive control system constitute personal demands able to boost the negative impact of job demands and foster the occurrence of workers’ burnout [47].

Moreover, empirical findings indicate that job resources and work engagement are not linked through a one-way relationship, but rather they mutually influence each other. Xanthopoulou and colleagues [48] revealed that job and personal resources exert a lagged effect on employees’ work engagement which, in turn, resulted in enhanced personal and job resources over time. Hence, engaged employees’ not only view themselves as being more optimistic and self-efficacious, but they also perceive enhanced levels of autonomy, supervisory coaching, performance feedback and opportunities for professional development in the workplace. In a similar vein, Schaufeli, Bakker and Van Rhenen [49] conducted a longitudinal study on a sample of managers and showed that engagement was predictive of improved job resources one year later, therefore an enhanced perception of social support, job autonomy, opportunities to learn and to develop, and performance feedback. Consistent to these findings, work engagement has been shown to have a positive, lagged effect on next week’s job resources (*i.e.*, autonomy, social support from colleagues, and exchange with supervisor) on a sample of teachers [50]. A three-way longitudinal study provided further support to this evidence by showing that engagement among teachers may positively affect the perception of the opportunities to learn and develop in the workplace and employees’ faculty to influence their work [51]. Overall, these results extend the motivational process postulated by the JD-R Model with concrete evidence that also work engagement may be conceived as an antecedent of the perceived job resources. In other words, the experience of a great level of engagement may enable employees’ to easily identify, trigger and even produce additional resources.

In contrast to work engagement, the negative form of working hard (*i.e.*, workaholism) seems to be associated to an increased perception of the requirement to carry out difficult and demanding work tasks. As previously stated, workaholic employees comply with their inner compulsion to work in order to prevent the tension, restlessness, and feelings of guilt and worthlessness that arise when they do not work [36]. As a consequence, these employees attempt to complete tasks more extensively than necessary not because their jobs require them to do so, but they actively strive in order to actively create more work for themselves [52]. Accordingly, the unreachable work standards set by workaholic employees translate into a greater difficulty in entrusting others with job responsibilities, and the unwillingness to delegate tasks to others [53].

In line with the research evidence summarized above, the current study represents an initial effort to extend the hypothesized association between trait affectivity and opposite types of working hard (see *Hypothesis 1* and *2*), by investigating the relationship between work engagement and job control, on the one hand, and between workaholism and job demands, on the other hand. Job control and job demands are key constructs within the Demands-Control Model [54], which constitutes a leading model in occupational health psychology. This model defines job demands as psychological stressors present in the work environment and entailing the requirement to carry out difficult and mentally demanding work with a high work pace, whereas job control refers to employees’ opportunity to be creative, participate in decision-making, and influence how they carry out their tasks.

Therefore, in the present research work engagement is expected to result in a higher level of perceived control over one’s job, due to the sense of energetic and effective connection with job experienced by these employees and the subsequent confidence on their ability to successfully control the surrounding environment [55]. In addition, workaholism is expected to foster the individual perception of the requirement to cope with demanding tasks and responsibilities (*i.e.*, job demands) since it may give reason for the necessity to dedicate an extraordinary amount of time to work [30]. Based on this rationale, the remaining hypotheses were formulated as follows:

*Hypothesis 3*. Work engagement mediates the relationship between positive affectivity and job control.

*Hypothesis 4*. Workaholism act as a mediator in the relationship between negative affectivity and job demands.

## 2. Methods

### 2.1. Procedure and Participants

The current research was conducted in nine general hospitals located in Northern Italy as part of an occupational health survey among healthcare workers. The medical director of each hospital was asked to hand out the questionnaires to the head physicians working in his staff. The questionnaire included a cover letter containing background information about the general aim of the study. In the introduction to the survey, participant anonymity was emphasized and confidentiality guaranteed. Accordingly, participants were instructed to place the filled-out questionnaires in a ballot box placed in each hospital department.

A total of 438 head physicians received the questionnaire, of which 269 returned the competed survey in the ballot box (response rate; 61.4%). Participants were mostly men (55.8%) and the mean age was 47.37 (SD = 8.87). Moreover, they worked in different hospital units: Emergency Medicine (23.8%); Internal Medicine (16.3%); Cardiology (15.6%); Maternity and Pediatrics (13%); General Surgery (12.6%); Pathology and Radiology (11.2%); Oncology (4.5%) and Mental Health Services (3%). The majority of the sample had a permanent job (82.5%), worked full-time (97.8%), and the mean tenure was 12.01 years (SD = 8.65).

### 2.2. Measures

*Positive Affectivity* and *Negative Affectivity* were assessed using the Positive and Negative Affect Scale (PANAS; [12]). A standard translation-back translation procedure was used for the Italian version of the questionnaire [56]. Participants’ trait affectivity was assessed by asking how often they experienced a particular effect on a scale ranging from 1 = not at all to 5 = very often. Positive affectivity included the following 10 items: Enthusiastic, Interested, Determined, Excited, Inspired, Alert, Active, Strong, Proud, and Attentive. Conversely, the 10 items concerning negative affectivity were: Scared, Afraid, Upset, Distressed, Jittery, Nervous, Ashamed, Guilty, Irritable, and Hostile. Cronbach’s alpha was α = 0.86 for positive affectivity and α = 0.90 for negative affectivity.

*Work Engagement* was assessed with the vigor and dedication subscales of the Italian version [57] of the short Utrecht Work Engagement Scale [58]. Since vigor and dedication represent the core dimensions of work engagement, the scores on these subscales, which each include three items, are considered as an indicator of work engagement [59]. Participants answered items such as: “At my work, I feel bursting with energy” (vigor) and “I am enthusiastic about my job” (dedication). All items were scored on a seven-point Likert scale that ranged from 0 = never to 6 = always. Cronbach’s alpha was α = 0.83 for Vigor, α = 0.87 for Dedication, and α = 0.88 for the total score on Work Engagement.

*Workaholism* was assessed using the Italian version [60] of the 10-item Dutch Work Addiction Scale [52]. This measure consists of two subscales composed of five items each: working excessively and working compulsively. Example items are: “I seem to be in a hurry and racing against the clock” (working excessively); “I feel obliged to work hard, even when it’s not enjoyable” (working compulsively). The response options varied on a four-point frequency scale ranging from 1 = never to 4 = always. The internal consistency of these scales was α = 0.74 for Working Excessively, α = 0.74 for Working Compulsively, and α = 0.79 for the total score on Workaholism.

*Job control* was measured with the scale taken from the Job Content Questionnaire ([61]; Italian version, [62]) and composed of nine items that assess the perceived degree of influence over one’s tasks. An example item is: “On my job, I am given a lot of freedom to decide how I do my work”. Participants were asked to indicate the extent to which they agree with each of the items, using a 4-point scale ranging from 1 = strongly disagree to 4 = strongly agree. Cronbach’s alpha for the scale was α = 0.65.

*Job demands* were assessed using the scale taken from the Job Content Questionnaire ([61]; Italian version, [62]) consisting of nine items that assess various psychological job demands such as mentally demanding work, conflicting work demands, and a high work pace. An example item is: “My job is characterized by conflicting demands”. Each item was answered on a four-point Likert scale from 1 (strongly disagree) to 4 (strongly agree). In the present study, the reliability of the scale was α = 0.67.

### 2.3. Strategy of Analysis

The hypotheses were simultaneously tested with structural equation modeling (SEM) using the AMOS 5 software package [63] and the maximum likelihood method of estimation. As previously mentioned, the key-components of vigor and dedication indicated the latent work engagement factor, whereas workaholism was included as latent factor indicated by the observed levels of working excessively and working compulsively. The remaining variables included single indicators equal to the average score of the corresponding scale, as the inclusion of redundant indicators provides less research benefit than single indicators of additional latent variables [64]. The error variance of these single indicator latent variables was computed by using the formula (1 − α)*σ^2^ [65]. In addition to the main mediation hypothesis, the final model included additional paths from positive affectivity to workaholism and from negative affectivity to work engagement. The mediation hypotheses were tested by comparing alternative models through Chi-square differences tests. The hypothesized model reporting exclusively the indirect paths was compared to a model including the direct effects from positive affectivity to job control and from negative affectivity to job demands. Besides the χ^2^ goodness-of-fit statistic, the models’ fit was assessed using the Tucker-Lewis Index (TLI), the Comparative Fit Index (CFI), the Goodness-of-Fit Index (GFI), and the Root Mean Square Error of Approximation (RMSEA). Generally, the fit is considered acceptable when the TLI, CFI and GFI are greater than 0.90 and the RMSEA is equal to or less than 0.08 [66]. In addition, the indirect relationships between positive affectivity and job control and negative affectivity and job demands were assessed through bootstrapping and Sobel tests. Bootstrapping is a statistical resampling method that generates an estimate of the indirect effect, including a 95% confidence interval. When zero is not included in the confidence interval, the null hypothesis that x has not an indirect effect on y through the proposed mediator can be rejected.

## 3. Results

### 3.1. Descriptive Statistics

The means, standard deviations, correlations, and internal consistencies for all study variables are presented in Table 1. Pearson’s correlations showed that all relationships between the study variables were significant in the expected direction.

### 3.2. Model Testing

**Figure 1:**
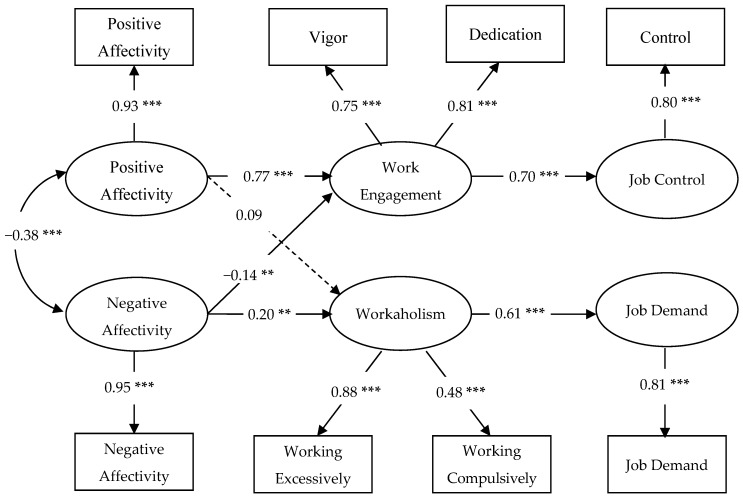
Standardized path coefficients of the full mediation model (M1). ** *p* < 0.01; *** *p* < 0.001; dotted lines denote non-significant effect.

As can be seen in the first row of Table 2, the proposed model (M1) showed an excellent fit to data, with a RMSEA < 0.06, and TLI, CFI and GFI indices exceeding 0.95 [67]. In this model, all indicators loaded significantly on their intended latent factors and all effects were in the expected direction. As displayed in Figure 1, results indicated that positive affectivity had a positive, direct relation with work engagement (β = 0.77, *p* = 0.000). This result fully supported *Hypothesis 1*. Moreover, negative affectivity was significantly and positively related to workaholism (β = 0.20, *p* = 0.01). This result provided evidence for *Hypothesis 2*. Moreover, the additional paths indicated that positive affectivity was not significantly related to workaholism (β = 0.09, *p* = 0.27). In contrast, negative affectivity showed a negative association to work engagement (β = −0.14, *p* = 0.02). In order to test whether work engagement and workaholism play a mediating role in the proposed model, M1 was compared with a partial mediation model incorporating two direct paths from positive affectivity to job control and from negative affectivity to job demands (M2). As reported in the second row of Table 2, the results indicated that the inclusion of these additional paths did not improve the model fit (Δχ^2^ (2) = 1.20, *ns*). Accordingly, the paths from positive affectivity to job control (β = −0.01, *p* = 0.93) and from negative affectivity to job demands (β = −0.08, *p* = 0.26) were non-significant.

In the next series of analysis, bootstrapping was used to assess the hypothesized mediation effects. The obtained results showed that the indirect effect of positive affectivity on job control through work engagement was significant. Indeed, the bias-corrected confidence interval (B-CCI) ranged from 0.097 to 0.699. Subsequent Sobel tests supported this result (z = 4.65, *p* = 0.000). Taken together, these results fully supported *Hypothesis 3*. The results of bootstrap analyses also indicated that the mediation effect of workaholism in the relationship between negative affectivity and job demands was significant, with a B-CCI ranging from 0.001 to 0.102. Again, the results of Sobel Test supported the significance of this indirect effect (z = 2.44, *p* = 0.01). Therefore, *Hypothesis 4* was supported as well.

## 4. Discussion

The present research was aimed at exploring individual characteristics that may explain differences in perception of job demands and job control among employees working in a healthcare setting. It represented a first attempt to delve into the role of affectivity as a personal trait predisposing employees towards different forms of heavy work investment, namely work engagement and workaholism.

Our findings supported the presence of a positive, direct relationship between positive affectivity (e.g., energy, enthusiasm, concentration) and work engagement, on the one hand, and a between negative affectivity (e.g., distress, nervousness, pessimism) and workaholism, on the other hand. This evidence is in line with previous studies indicating a strong association between positive and negative effects and work engagement and workaholism, respectively [68]. Accordingly, employees characterized by positive affectivity have larger individual thought-action repertoires, more innovative behaviors, more energy and motivation, a better relational and social functioning, and more personal resources, thus they display an enhanced level of engagement, professional development and success [69]. In contrast, employees characterized by negative affectivity are worried about their job, feel guilty, anxious and dissatisfied [12]. Consequently, they are likely to develop an addiction to work (*i.e.*, workaholism) and tend to work compulsively in order to counteract the negative affectivity that would emerge if they are not working [70]. Furthermore, the inclusion of additional paths revealed that negative affectivity is related to decreased levels of work engagement. This evidence is not unexpected, given the presence of initial results suggesting an association between negative emotions and engagement [71]. On the other hand, the results of early investigations on this topic refer to affective states rather than enduring dispositional traits.

Overall, the current results indicate that employees with a strong positive affectivity are more engaged at work and perceive a higher level of control on working events [72]. In contrast, employees reporting a strong negative affectivity are more likely to become workaholic and create more job demands for themselves, because they are highly perfectionist, obsessed with unattainable standards, reluctant in delegating responsibilities to their colleagues, and inclined to spend a lot of time and efforts in unimportant activities [73]. Wojdylo *et al.* [42] suggested that workaholic employees, like other types of addicts (e.g., gamblers), experience a sense of craving, a subjective state of high-urge to act—in this case to work—with the aim of compensating negative emotions through performance-conditioned self-worth and neurotic perfectionism. In particular, working hard offers illusorily, positive incentives (feelings of efficiency and self-worth) and relief from negative emotions. Workaholic employees are driven by their desire to compensate for a low level of perceived self-worth [74], and to escape from negative emotions and feelings of inadequacy. Thus, it may be concluded that their perception of the work environment is strongly conditioned by the evidence that this sphere of life may shape their sense of self-confidence, and “psychological safety”.

The current results also agree with previous research suggesting that positive affectivity is related to the development of personal resources such as coping strategies and the individual repertoire of cognitive and behavioural responses [70]. Indeed, engaged employees exhibit a stronger commitment to work, take adequate decisions, help their colleagues, and cope better with demanding situations at work. Moreover, the present study emphasized the role played by dispositional features of employees, in particular trait affectivity, in interpreting and reacting to a demanding and stressful work environment, such as the healthcare setting. In particular, physicians have to deal with several job demands, such as high levels of responsibility, conflictual relationships with colleagues and nursing staff, emergency situations, changing schedules, demanding patients, and high workload [75,76].

### 4.1. Study Limitations

The main limitation of the current study is its cross-sectional nature precludes the assessment of cause—effect relationships. A rigorous longitudinal research design would reduce the likelihood of the findings having arisen due to chance and would allow us to investigate whether the current results remain stable over time. The existing literature may also suggest alternative models that conceptualize the hypothesized relationships differently. However, an alternative model in which job characteristics mediate the relationship between affectivity and employee well-being showed a poor fit to the data, with χ^2^ (df = 17) = 66.97; *p* = 0.000, TLI = 0.84, CFI = 0.90, and RMSEA = 0.11. Moreover, in the current study the scales taken from the Job Content Questionnaire [61] and aimed to assess job control and job demands had a reliability coefficient slightly lower than the criterion of 0.70, which is traditionally considered as a rule of thumb [77]. However, this value is not universally accepted. For instance, DeVellis [78] proposed 0.65 as a minimum threshold for an acceptable coefficient α. Nunnally [79] stated that even scales with an internal consistency higher than 0.60 can be used for research purposes. In addition, the current data set was drawn from a very specific group of employees (*i.e.*, physicians), thus decreasing the opportunity to generalize the obtained results to the entire working population. Further investigations should replicate the present results on employees pertaining to different occupations. In addition, the current study did not include objective measures of the work environment that could have provided even more substantial support for our hypotheses. On the other hand, data were collected on a sample of head physicians working in different hospitals but all located in the same Italian region and managed by a single organization. Hence, it can be argued that they were exposed to comparable work environments and conditions and that the current findings can primarily be ascribed to differences in dispositional traits, *i.e.*, trait affectivity, that fosters the level of employees work engagement and workaholism.

### 4.2. Practical Implications

The current results emphasized the role of trait affectivity as antecedent of work engagement and workaholism that, in turn, are related to enhanced levels job control and job demands. Accordingly, the perception of job control and demands in the workplace is not merely a function of organizational characteristics, but rather it reflects stable individual differences in trait affectivity. This evidence is consistent with previous findings suggesting that organizational environments that promote positive affectivity among employees would take advantage of beneficial outcomes such as an increased level of creativity [80], organizational citizenship behaviors [81], and an enhanced task performance [73]. In contrast, negative affectivity may lead to detrimental consequences for organizations, such as an impaired task performance, recurrent counterproductive and withdrawal work behaviors, and an increased rate of occupational injuries [82]. Hence, a thorough understanding of employees’ dispositional traits, *i.e.*, affectivity, would allow organizations to develop effective training and motivational interventions aimed at improving work engagement levels and discouraging maladaptive conducts such as workaholism [11]. To this purpose, organizations should implement appropriate job design strategies aimed at facilitating positive work events that are likely to evoke the experience of positive affective responses [83].

Traditionally, dispositional traits are conceived as stable characteristics that can hardly be adjusted in order to guarantee a suitable fit between new employees and their work role [84]. However, research evidence based on longitudinal data suggests that these traits are less stable than other dispositional characteristics, such as the Big 5 traits [85]. Therefore, intervention strategies could effectively be focused on affectivity in order to help employees to increase their self-knowledge and self-awareness. Through a deeper understanding of themselves employees could be enabled to change their workplace attitudes and, as a consequence, alter their job involvement and the perceived level of job control and job demands. In particular, the ability to self-regulate emotions may represent a good starting point for interventions aimed at decreasing workaholism and fostering work engagement. Workaholic employees are characterized by low self-relaxation competencies, thus a low ability to self-regulate negative emotions, whereas engaged employees report high self-motivation competencies, hence they are particularly able to self-regulate positive emotions that translate into a strong identification with their work goals [86].

In conclusion, the present results may contribute to the development of effective interventions designed to help employees characterized by negative affectivity to reduce their tendency to work as an antidote and, in turn, the perceived requirement to carry out difficult tasks with a high work pace (*i.e.*, job demands). At the same time, the improvement of self-motivation competencies may lead employees to experience a positive type of involvement in their work and, consequently, to perceive a considerable autonomy in performing their work tasks (*i.e.*, job control).

## 5. Conclusions

The current results indicate that positive affectivity is related with work engagement, which, in its turn, showed a positive association with job control. In contrast, workaholism mediated the relationship between negative affectivity and job demands. Thus, it may be concluded that affectivity influences indirectly the interpretation and perception of the work environment. Overall, this research represents an initial attempt to consider simultaneously dispositional characteristics and the meditational role of work engagement and workaholism in explaining different perceptions of job demands and resources.

## Figures and Tables

**Table 1:** Means, Standard deviation, Cronbach’s Alphas (in brackets), and Correlations among the study variables (*N* = 269).

			*r*									
	***M***	***SD***	**1**	**2**	**3**	**4**	**5**	**6**	**7**	**8**	**9**	**10**
1. Positive Affectivity	3.79	0.56	(0.86)									
2. Negative Affectivity	1.65	0.63	−0.34 ***	(0.90)								
3. Vigor	4.49	1.16	0.57 ***	−0.32 ***	(0.83)							
4. Dedication	4.98	1.06	0.61 ***	−0.33 ***	0.60 ***	(0.87)						
5. Work Engagement	4.73	0.99	0.66 ***	−0.37 ***	0.90 ***	0.89 ***	(0.88)					
6. WE	2.70	0.54	0.01	0.13 *	−0.09	−0.03	−0.07	(0.74)				
7. WC	2.10	0.59	0.10	0.16 *	0.05	−0.01	0.02	0.43 ***	(0.74)			
8. Workaholism	2.40	0.48	0.07	0.16 **	−0.03	−0.02	−0.03	0.83 ***	0.43 ***	(0.79)		
9. Job control	3.31	0.31	0.43 ***	−0.19 **	0.42 ***	0.47 ***	0.49 ***	−0.08	0.83 ***	−0.08	(0.65)	
10. Job demands	3.03	0.37	0.02	0.13 *	−0.07	−0.02	−0.05	0.44 ***	−0.08	0.37 ***	0.03	(0.67)

Notes: WE = Working Excessively; WC = Working Compulsively; * *p* < 0.05; ** *p* < 0.01; *** *p* < 0.001.

**Table 2:** Goodness-of-Fit indices of the nested models and tests of indirect relationships (*N* = 269).

Model	χ^2^	df	TLI	CFI	GFI	RMSEA	Model Comparison	Δχ^2^	Δdf
M1. Hypothesized Model	25.02	17	0.97	0.98	0.97	0.04	-	-	-
M2. Partial Mediation Model	23.82	15	0.96	0.98	0.97	0.05	M1 − M2	1.20	2

Notes: χ^2^ = Chi-square, df = degrees of freedom; CFI = Comparative Fit Index; TLI = Tucker-Lewis Index; GFI = Goodness-of-Fit Index; RMSEA = Root Mean Square Error of Approximation; Δ = difference test.
